# Biosensor-guided detection of outer membrane-specific antimicrobial activity against *Pseudomonas aeruginosa* from fungal cultures and medicinal plant extracts

**DOI:** 10.1128/spectrum.01536-23

**Published:** 2023-10-26

**Authors:** Steve Shideler, Tyson Bookout, Azka Qasim, Lauren Bowron, Qiaolian Wu, Kangmin Duan, Roland Treu, Shauna Reckseidler-Zenteno, Shawn Lewenza

**Affiliations:** 1 Department of Microbiology, Immunology, and Infectious Diseases, University of Calgary, Calgary, Alberta, Canada; 2 College of Life Sciences, Northwest University, Xian, China; 3 Department of Oral Biology, University of Manitoba, Winnipeg, Manitoba, Canada; 4 Faculty of Science and Technology, Athabasca University, Athabasca, Alberta, Canada; University of Manitoba, Winnipeg, Manitoba, Canada

**Keywords:** *Pseudomonas*, biosensors, outer membrane damage, polymyxin resistance, antibiotic discovery, natural products, PhoPQ, PmrAB

## Abstract

*Pseudomonas aeruginosa* responds to sub-lethal antimicrobial exposure by inducing the expression of lipopolysaccharide (LPS) surface modifications that mask antibiotic binding sites and contribute to repair and resistance of the outer membrane (OM). We exploit these membrane damage-responsive operons in a biosensor approach used to discover new antimicrobials that specifically target the OM. Chromosomal transcriptional *luxCDABE* reporters from the *pmr* (polymyxin resistance; aminoarabinose LPS modification) and *speD2E2* (spermidine synthesis) operons are induced by validated outer membrane-acting agents including cationic antimicrobial peptides, cation chelators, ascorbic acid, detergents, and cell wall synthesis inhibitors cycloserine and bacitracin. To identify novel sources of OM-disrupting antimicrobials, we used these OM damage-responsive biosensors to screen a panel of fungal culture supernatants for novel antimicrobial and biosensor activity. Biosensor activity was used to determine the optimal time point of antimicrobial production from fungal supernatants and to guide the purification of active fractions after size-exclusion chromatography. Water and ethanol extracts of Chinese medicinal plants also proved to be a source of biosensor activity. The pathogen box is a 400-member drug library of potential antimicrobials, but none of these compounds induced our OM damage biosensors. This novel, sensitive, cell-based screening assay has potential for future discovery of lead compounds that specifically target the outer membrane, which is a significant barrier to antibiotic entry into Gram-negative bacteria.

**IMPORTANCE:**

New approaches are needed to discover novel antimicrobials, particularly antibiotics that target the Gram-negative outer membrane. By exploiting bacterial sensing and responses to outer membrane (OM) damage, we used a biosensor approach consisting of polymyxin resistance gene transcriptional reporters to screen natural products and a small drug library for biosensor activity that indicates damage to the OM. The diverse antimicrobial compounds that cause induction of the polymyxin resistance genes, which correlates with outer membrane damage, suggest that these LPS and surface modifications also function in short-term repair to sublethal exposure and are required against broad membrane stress conditions.

## INTRODUCTION

The *pmr* operon (*PA3552-PA3559; arn*) is responsible for the addition of aminoarabinose to lipid A of *Pseudomonas aeruginosa*, which serves to mask the negative charges of the core lipid A phosphates and protect against antimicrobial peptide killing (1 – 3). The spermidine synthesis genes *speD2E2* (*PA4773-PA4774*) produce the polycation spermidine (+3 charge) that is surface localized and protects the outer membrane (OM) from antimicrobial peptide damage, likely by masking negative surface charges (4). Mini-Tn*5-luxCDABE* mutagenesis was previously carried out to create a library of transposon insertion mutants that also function as transcriptional *lux* fusions (2), which were used in the following studies to describe the conditions where these genes were induced. Both operons were originally reported to be induced under Mg^2+^-limiting conditions and controlled by the Mg^2+^-responsive PhoPQ and PmrAB two component systems (2, 5, 6). Magnesium cations bind to and stabilize the outer surface of LPS (7), and these modifications act as substitutes for metal cations when they are unavailable. In conditions that promote the expression of these operons, *P. aeruginosa* displays a pronounced antibiotic resistance, and conversely, mutations in these genes result in antibiotic sensitivity phenotypes to antimicrobial peptides and aminoglycosides (2, 4, 8 – 10).

The *pmr* and *spe* transcriptional *lux* fusions were next shown to be induced by exposures to sub-minimal inhibitory concentration (MIC) levels of a range of polymyxins and numerous antimicrobial peptides (6, 11), independent of the Mg^2+^-responsive PhoPQ and PmrAB two-component systems (11, 12). The ParRS and CprRS two-component systems are required for induction of the *pmr* and *spe* operons by a broad panel of antimicrobial peptides and structures, and the nature of the specific signal detected by *P. aeruginosa* has not been clearly identified (11, 12). It is generally thought that the ParS and CprS sensors detect unique antimicrobial peptides in *P. aeruginosa*, and CprS has a highly negative sensing domain, similar to *Salmonella* PhoQ, which senses antimicrobial peptides directly (11). We also demonstrated that extracellular DNA is an efficient cation chelator and bacterial membrane disrupter that induces the expression of these chromosomal transcriptional reporters (4, 9). Consistent with their regulation by exogenous eDNA, these outer surface modification systems are also induced by the DNA component of neutrophil extracellular traps (NETs) (13). Extracellular DNA and the DNA within NETs both have cation chelating, antimicrobial activity that requires direct contact and the phosphates of DNA (9, 13). DNA chelates surface cations and causes major disruption of the outer and inner membranes (OM/IM), resulting in the release of outer membrane vesicles and membrane debris, which results in a rapid and lytic bacterial killing (9). Mutants in the *pmr* and *spe* operons are more sensitive to killing by neutrophil extracellular traps, indicating these DNA-induced modifications also protect against DNA killing (13).

While the *pmr* and *spe* genes are generally viewed as antibiotic (adaptive) resistance or immune evasion mechanisms, their induction by multiple threats to the outer membrane suggests that they may generally function as repair processes against OM-damaging agents. Therefore, we propose here that the *pmrF::lux* and *speE2::lux* transcriptional *lux* reporters will serve as effective biosensors for the discovery of new antimicrobial compounds that specifically damage the outer membrane. We describe a high-throughput screen of a panel of cell-free fungal supernatants, Chinese medicinal plant extracts, and biosensor-guided fractionation to demonstrate the potential for discovering novel antimicrobials that target the OM.

## MATERIALS AND METHODS

### Growth conditions

*P. aeruginosa* biosensor cultures were grown in Luria broth (LB), and fungal cultures were grown in Sabouraud Dextrose Broth (SDB) and on Sabouraud Dextrose Agar (SDA) (BIFCO). Stock solutions of antimicrobials were diluted into LB at concentrations two- to fourfold lower than their minimal inhibitory concentration values to promote a strong biosensor response at sublethal exposures. Twenty-nine fungal strains (Table 1) were obtained from the Athabasca University Biocollection (AUB) as frozen agar slants and used to inoculate SDA petri plates. The plates were then stored in bags with a moist paper towel and incubated at 25°C. Once sufficient fungal growth occurred, 15-mL test tubes were filled with 3 mL of SDB, and a sterile 10-µL pipette tip was used to cut a small agar core from the stock SDA fungal culture and incubated at 25°C until a fungal pellicle or sizable aggregates formed in the tubes. The MIC values of all the OM disrupting compounds used in this study are shown in Table 2.

**TABLE 1:** Names of medicinal plants and fungal isolates used in this study

Medicinal plants		Fungal isolates
Latin name	Chinese name	
*Andrographis paniculata* (Burm.f.）Nees.	穿心莲	*Agaricus sylvicola*
*Anemarrhena asphodeloides* Bge.	知母	*Armillaria* sp.
*Artemisia annua* L.	青蒿	*Bjerkandera adusta*
*Artemisia argyi* levl. et Vant	艾叶	*Clavicorona pyxidata*
*Chrysanthemum indicum* L.	野菊花	*Climacocystis borealis*
*Dictamnus dasycarpus* Turcz.	白鲜皮	*Climacodon septentrionalis*
*Forsythia suspense* (Thumb.) Vahl.	连翘	*Coriolopsis gallica*
*Gardenia jasminoides* Ellis.	栀子	*Crepidotus mollis*
*Gentiana manshurica* Kitag.	龙胆	*Flammulina fennae*
*Houttuynia cordata* Thunb.	鱼腥草	*Fomes fomentarius*
*Isatis indigotica* Fort.	大青叶	*Fomitopsis cajanderi*
*Lophatherum gracile* Brongn.	淡竹叶	*Ganoderma applanatum*
*Lycium chinense* Mill.	地骨皮	*Gloeophyllum trabeum*
*Paeonia suffruticosa* Andr.	牡丹皮	*Gymnopus dryophilus*
*Patrinia villosa* Juss.	败酱草	*Haploporus odorus*
*Phragmites communis* Trin	芦根	*Hericium coralloides*
*Prunus mume* (Sieb.）Sieb. et Zucc.	乌梅	*Hohenbuehelia* sp.
*Pulsatilla chinensis* (Bge.) Regel.	白头翁	*Hypsizygus tessulatus*
*Scutellaria baicalensis* Georgi.	黄岑	*Inonotus rheades*
*Scutellaria barbata* D. Don	半枝莲	*Lentinula edodes*
*Smilax glabra* Roxb.	土茯苓	*Lentinus lepideus*
*Sophora flavescens* Ait.	苦参	*Lenzites betulina*
*Viola Yedoensis* Makino.	紫花地丁	*Marasmius oreades*
		*Mycena pura*
		*Phaeomarasmius erinaceus*
		*Phellinus pini*
		*Pleurotus populinus*
		*Trametes pubescens*
		*Trametes suaveolens*

**TABLE 2:** MIC values of OM-disrupting compounds against PAO1 grown in LB media

Compound	MIC
Ascorbic acid	5 mg/mL
Bacitracin	10 mg/mL
Bile Salts	2 mg/mL
Colistin	0.5 ug/mL
CTAB	1 mg/mL
D-Cycloserine	1 mg/mL
Deoxycholate	> 2 mg/mL
DNA	5 mg/mL
EDTA	300 ug/mL
Polymyxin B	> 1 ug/mL
SDS	>0.005%

### Fungal supernatant harvesting

After the desired incubation period, a 1,000-µL sterile pipette was used to harvest the supernatant from the test tubes. The supernatant was transferred to 1.5 mL sterile Eppendorf tubes and centrifuged at 13,200 rpm for 10 minutes at 25°C. An 18-gauge sterile syringe (10 mL volume) was used to extract the supernatant and avoid disturbing the cell pellet that formed at the bottom of the tube, which were then sterilized using a 0.2 µM cellulose acetate filter. Cell-free supernatants were stored in 1-mL aliquots in sterile Eppendorf tubes and placed in 4°C for storage. Fungal supernatants were further concentrated 2× using a vacufuge at 30°C for 2 hours to reduce the volume.

### Ninety-six-well microplate biosensor screening

Overnight LB cultures of *speE2:lux* and *pmrF::lux* were diluted 1:10 in LB. Ten microliters of diluted overnight cultures were added to 80 µL of LB and 10 µL of concentrated, sterile, fungal supernatant (10% vol/vol) or to 70 µL of LB and 20 µL of fungal supernatant (20% vol/vol). For negative controls, 10 µL of diluted overnight biosensor cultures was added to LB with 10–20 μL of sterile SDB, to control for any influence of SDB on gene expression. All supernatant samples were tested in triplicate. Before starting the assay, 60 µL of mineral oil was added to each microplate well to prevent the wells from drying. The microplate was then loaded into a Victor^3^ 1420 Multilabel Counter (PerkinElmer), and growth (OD_600_) and luminescence (CPS) were measured every 20 minutes throughout 15 hour at 37°C.

### NPN uptake assay to measure outer membrane permeability

Overnight cultures of PAO1 were subcultured 1:50 to fresh LB media and grown to an OD_600_ of 0.5 at 37°C. Cultures were then centrifuged at 8,000 rpm for 3 minutes, and cells were resuspended in equal volumes of 5 mM HEPES (pH 7.2) containing 5 mM glucose. Cells were pretreated with sodium azide (0.2%) to disable active efflux. 1-N-phenylnaphylamine (NPN) is a fluorescent dye when integrated into the hydrophobic environment of bacterial membranes. NPN was added at a final concentration of 0.01 mM to measure both the baseline fluorescence and the OM disruption by antimicrobial compounds and increase in NPN uptake (14). After NPN addition, cells were treated with antimicrobials at ~5 seconds, and fluorescence was measured for 60 seconds using a Spectra Max M2 spectrophotometer using the SoftMax Pro 6 software. The settings for green excitation and emission spectra were set to 350 nm and 420 nm, respectively.

### Propidium iodide uptake to measure inner membrane permeability

PAO1 cultures (5 mL) were grown in LB to mid-log phase (~3.5 hours). Cells were pelleted by centrifugation (8,000 rpm, 3 minutes), and the pellet was washed once and resuspended in 5 mL phosphate buffered saline, and propidium iodide (PI) was added to a final concentration of 5 µg/mL. Aliquots of 100 µL were dispensed to a black, clear bottom microplate. Antibiotics were added at the concentrations indicated and were compared with untreated cells by measuring red fluorescence every 20 minutes as an indicator of PI uptake into the cell after disruption of the OM and IM (15, 16) (Ex 555/38, Em 632/45) in the Victor^3^ 1420 Multilabel Counter (PerkinElmer).

### Protein fractionation of fungal culture supernatants

One liter Erlenmeyer flasks containing 600 mL of Sabouraud Dextrose Broth were inoculated with five 1 cm^3^ agar chunks from stock fungal culture and incubated in a 25°C. Cultures were removed after 6–16 weeks of growth using a 25-mL pipette to extract the supernatant from the flasks. The supernatant was then centrifuged at 13,000 rpm for 10 minutes and then filtered using a 0.2-µM Nalgene Super Mach vacuum filter. Supernatants were passed through the Amicon <10 kD cut-off filter systems to remove large proteins. The resulting supernatant was put on ice and stirred while slowly adding ammonium sulphate and stirred for 4 hours on ice. The flask was held overnight at 4°C and then centrifuged for 2 hours at 4,000 rpm to collect the precipitated proteins. After carefully removing the supernatant, the pellet was resuspended in 50 mM ammonium bicarbonate buffer. The resuspended pellet was then placed in a 1-kD cut-off dialysis tube and placed in a beaker filled with 80 mL of ammonium bicarbonate buffer for dialysis for 24 hours. Dialysis was repeated for another 24 hours with fresh buffer. The solution was removed from the dialysis bag, and 3 mL was concentrated in a vacufuge, down to 700 µL. Gel filtration chromatography was used to further purify the fungal supernatant proteins using FPLC and a Superdex 200 10/300 gel filtration column. Fractions were collected in 48 wells of a deep 96-well microplate, each containing 500 µL, for a total volume of 24 mL. Fifty millimolars ammonium bicarbonate buffer was used during the column filtration.

### Biosensor screening for activity within fungal protein fractions

All fractions acquired from the gel filtration chromatography were then retested in 96-well plate format with the *pmrF::lux* biosensor strain, for identifying the active fractions that induce gene expression. The control well contained 80 µL of LB and 20 µL of 50 mM ammonium bicarbonate solution (fractionation buffer). Sample wells contained 70 µL of LB, 10 µL of 1/10 diluted biosensor, and 20 µL of fractionated supernatant. The plates were then run in the Victor^3^ overnight using the previously described OD_600_/CPS 15 hours protocol.

### Protein identification

Active fractions were run on an SDS-PAGE gel, and bands of interest were excised and sent for mass spectrometry analysis (LC-MS-MS) at the Southern Alberta Mass Spectrometry (SAMS) Centre or the Proteomics Platform of the Quebec Genome Center. A synthetic peptide (RADDTTVLSASGPGRN) derived from a fungal glycoside hydrolase was synthesized by GenScript for subsequent experiments.

### Chinese medicinal plant extracts and biosensor screening

A series of ethanol or water extracts were prepared as previously described (17) from the 23 Chinese medicinal plants listed in Table 1. Total protein concentrations in the extracts were determined and added to LB at cultures up to 3 mg/mL for gene expression assays, as described above. The positive control was the addition of sublethal amounts of polymyxin B (PxnB) (<1 µg/mL), and negative controls had nothing adding to the biosensor cultures. The extracts were tested before, and after filtration through ultracentrifugation, filters with 10 kD and 1 kD cut-offs (compounds with molecular weight > 1 kD and <10 kD in the samples) were tested.

### Pathogen box screening

The pathogen box is a collection of 400 drug-like compounds that are active against bacterial pathogens and other neglected infectious diseases (18). This library was provided in five microplates, with 10 µL of each compound dissolved at 10 mM in dimethyl sulfoxide. To screen this library, we added 1 µL of each compound into 100 µL LB media, giving a final concentration of 100 µM. All 400 compounds were screened once against each of the *pmrF::lux* and *speE2::lux* biosensors, in search of compounds that induced expression of these OM repair genes. Expression was compared with and normalized to control cultures with no added drugs, and the gene expression values from 2 hours 45 minutes of exposure were reported.

## RESULTS

### Known outer membrane-damaging agents induce the expression of OM protection and repair systems

**Fig 1:**
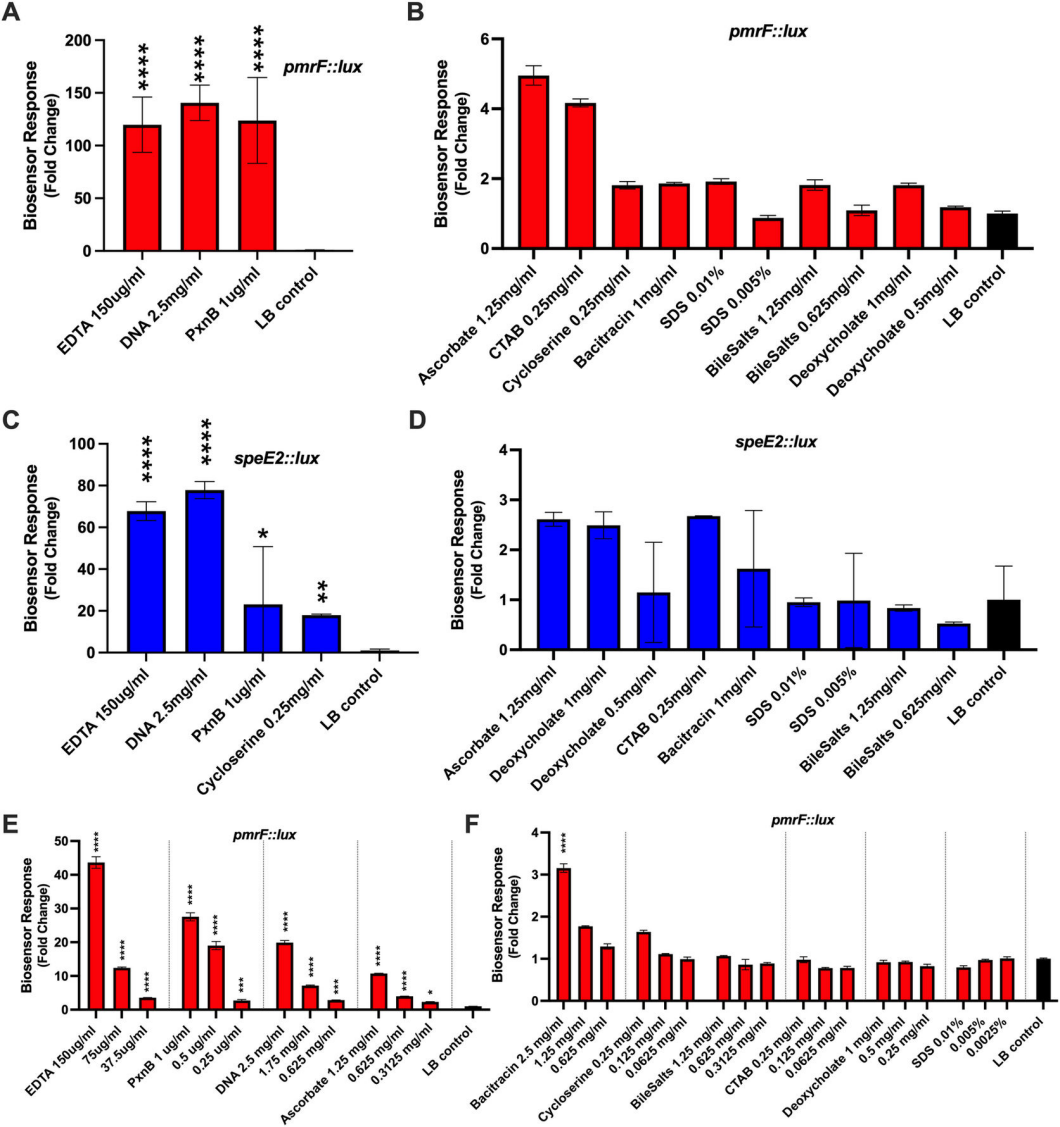
Outer membrane-acting compounds induce expression of *pmrF::lux* and *speE2::lux* biosensors and damage the outer membrane at sub-lethal concentrations. Strong (**A and C**) and weak (**B and D**) inducers of the *pmrF::lux* and the *speE2::lux* biosensors were detected in liquid LB cultures. To assess dose-dependent gene expression effects, each compound was tested at three different concentrations against the *pmrF::lux* biosensor (**E and F**). The values shown are the mean and standard error values of triplicates from the 7-hour time point of maximum expression. Compounds were tested at maximum sub-lethal concentrations, generally within two- to fourfold of the MIC (Table 2), where maximal *lux* responses were observed. All experiments were repeated at least five times. Gene expression was normalized to growth (CPS/OD_600_), and the fold change was determined by dividing the gene expression in the presence of the compound by expression without treatment. Significant differences compared with the LB control were determined by one-way ANOVA with Dunnett’s multiple comparisons post-test (****P < 0.0001, ***P < 0.001, **P < 0.005, and *P < 0.05).

The *pmr* and *speD2E2* chromosomal *lux* transcriptional reporters were previously shown to be induced by sublethal exposure to numerous types of antimicrobial peptides (6, 12), as well as chelators such as DNA and ethylenediaminetetraacetic acid (EDTA) (9). Chelators remove the stabilizing surface cations causing significant disruptions to the cell envelope, while antimicrobial peptides displace cations and disrupt the permeability of lipid bilayers, both of which cause rapid lysis and killing. Here, we wanted to determine the range of OM damage detection by our biosensors after challenge with diverse compounds that are known to disrupt the outer membrane. We identified additional compounds that induced both the *pmrF::lux* and *speE2::lux* biosensors (Fig. 1). Using the *pmrF::lux* sensor, the strongest induction was from the chelators EDTA and DNA and polymyxin B (PxnB) (Fig. 1A) but weakly induced by ascorbic acid and cetyltrimethylammonium bromide (CTAB) (>4-fold) (Fig. 1B). Ascorbic acid (vitamin C) permeabilizes the OM potentially due to its weak cation chelating activity (19). Detergents generally interact with, solubilize, and disrupt lipid bilayers, and CTAB is a cationic detergent whose specific mechanism of membrane disruption is poorly characterized (20). The detergents sodium dodecyl sulfate and bile salts caused low induction levels of *pmrF::lux* (~2-fold) (Fig. 1B). The *speE2::lux* biosensor also responded strongly to chelators, polymyxin B, and cycloserine (Fig. 1C) but weakly responded to ascorbic acid, deoxycholate, and CTAB (>2-fold) (Fig. 1D).

In addition to testing individual concentrations approaching the MIC values (Table 2), we also tested a range of sublethal concentrations to demonstrate the dose-dependent responses of the *pmrF::lux* biosensor to these compounds. Chelators, polymyxin B, ascorbate, and bacitracin all caused significant dose-dependent responses, where there was more gene expression with increasing concentrations of compounds (Fig. 1E and F). The dose-dependent gene expression responses likely represent greater amounts of membrane damage as the antibiotic concentration increases. Cycloserine inhibits peptidoglycan synthesis enzymes that ultimately disrupts cell wall integrity. Although not a classic OM disrupter, cycloserine was reported to cause envelope stress in *P. aeruginosa* leading to activation of *algU* and an increase in the production of outer membrane vesicles (21). Bacitracin is a polypeptide antibiotic that inhibits cell wall synthesis but is mainly used against Gram-positive bacteria and some Gram-negative bacteria.

### Biosensor activity generally correlates with the ability to disrupt the outer and inner bacterial membranes

**Fig 2:**
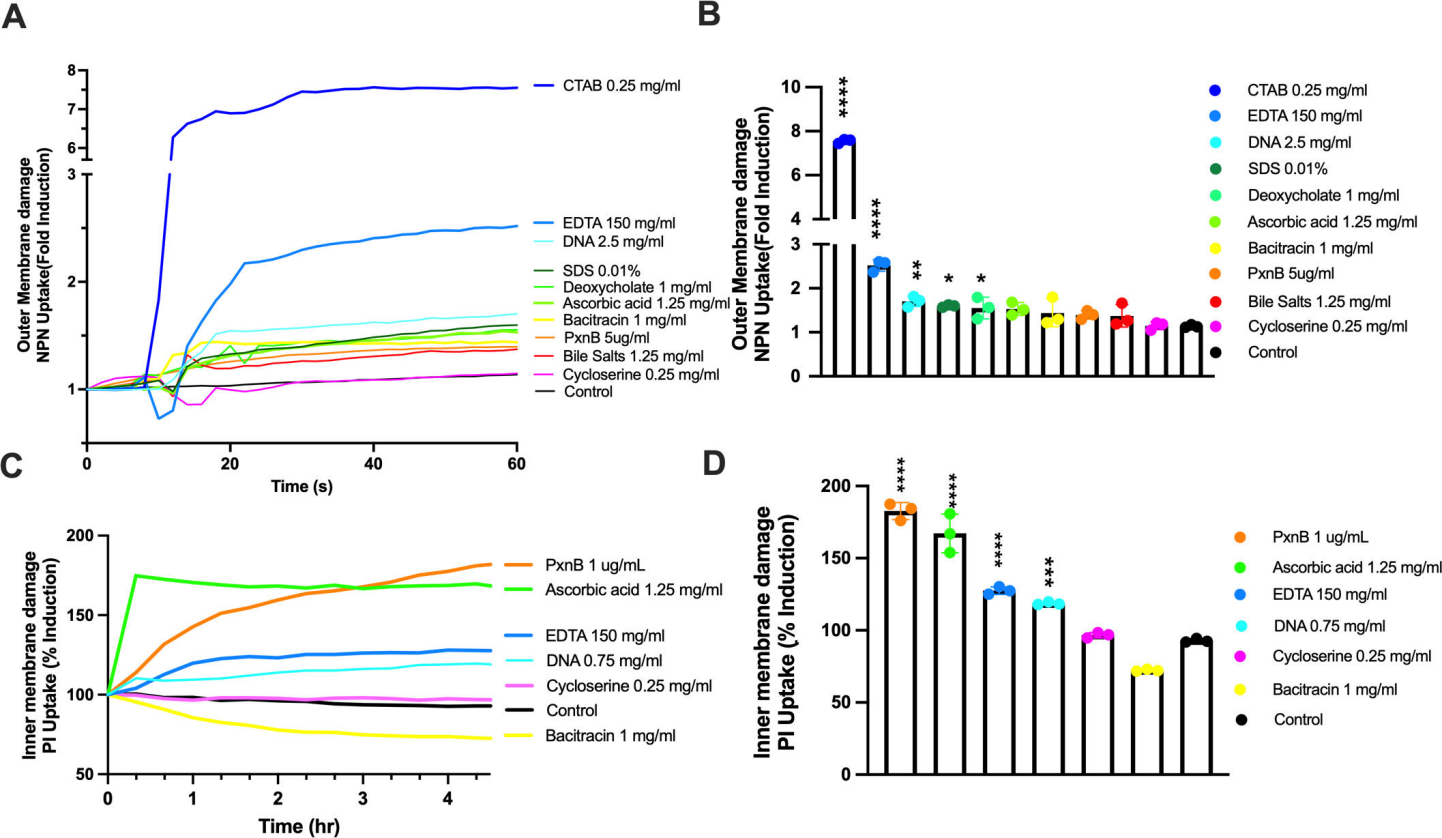
NPN uptake as an indicator of OM disruption and PI uptake as an indicator of IM disruption. (**A**) Baseline NPN fluorescence is recorded in the first few seconds, and then, the addition of OM-disrupting compounds causes a general increase in NPN fluorescence, as it integrates into the hydrophobic outer membrane after treatment. (**B**) The amount of NPN uptake after 60 seconds of treatment by a panel of compounds. (**C**) PI uptake was measured in 96-well microplates after treatment with outer membrane-damaging compounds. (**D**) The amount of PI uptake after 200 minutes of treatment. The concentrations used in panels A and B were similar to panels C and D. All experiments were repeated at least three times. The values shown are the mean and standard error values of triplicates, which were normalized to the time zero values. Significant differences compared with the untreated controls (black) were determined by one-way ANOVA with the Dunnett’s multiple comparisons post-test (****P < 0.0001, ***P < 0.001, **P < 0.005, and *P < 0.05).

To confirm that the biosensor activity corresponds to OM damage, we used the traditional outer membrane permeability assay that monitors the fluorescence and uptake of the compound NPN that is fluorescent when entering the hydrophobic environment of the outer membrane upon disruption (14). The baseline of NPN fluorescence is measured for 5 seconds, and then, the OM disrupting compounds are added, resulting in a rapid increase in NPN fluorescence that occurs with 20–40 seconds (Fig. 2A). We grouped the effects of these compounds into strong OM disrupters (CTAB, chelators, SDS, and deoxycholate) or weak OM disrupters that included polymyxin B, ascorbic acid, and bacitracin (Fig. 2A and B).

We also performed a propidium iodide uptake assay, which is used as an indicator of IM damage, allowing for PI binding and staining of DNA in the cytoplasm. PI is commonly used as the “dead” stain in live/dead staining of bacteria, as it enters dead cells and stains DNA. However, viable cells can also stain with PI, which can be isolated and grown after replating (15). Therefore, PI staining of bacteria is also an indicator of compromised OM and IM integrity. Most compounds from Fig. 1 were tested for the ability to promote PI uptake in wild-type *P. aeruginosa* PAO1 (Fig. 2). Polymyxin B and ascorbic acid caused the most rapid PI uptake, although this process occurred over the course of hours, compared with seconds for NPN uptake (Fig. 2A and C). Chelators also caused PI uptake and IM disruption, although cycloserine and bacitracin did not (Fig. 2D). In these experiments, we confirmed that this panel of compounds has a range of OM and IM disruption abilities, where in general, there was agreement between biosensor responses (Fig. 1) and outer membrane disruption (Fig. 2). Cycloserine was one exception, which was detected by the *speE2::lux* biosensor (Fig. 1C) but did not disrupt the OM or IM (Fig. 2). Bacitracin was only detected by the *pmrF::lux* (Fig. 1) and caused weak NPN uptake but not PI uptake (Fig. 2). Note that the biosensors demonstrated a wide dynamic range of detection, with fold changes greater than 100-fold, while the NPN and PI assays had a very narrow range of detecting membrane disruption and lower fold changes (Fig. 2). This result suggests that the biosensors are more sensitive to detecting OM damage than the NPN uptake assay.

### Visual detection of the luminescent biosensor responses to outer membrane-damaging compounds

**Fig 3:**
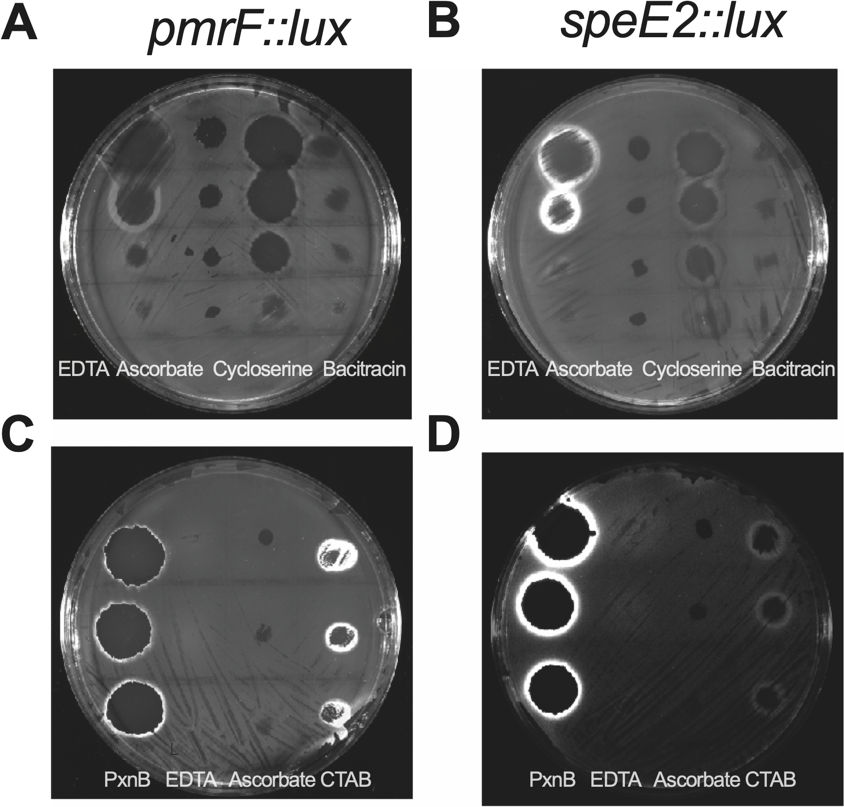
Rings of biosensor *lux* responses at the edges of killing zones by known outer membrane-acting compounds. All compounds were spotted in increasing amounts onto LB agar plates after spreading the surface with the *pmrF::lux* (**A and C**) or *speE2::lux* (**B and C**) reporter strains. Luminescent rings were visualized at the edges of the zone of killing for most compounds. For the top images (**A and B**), compounds were spotted in the following ranges: EDTA (50, 100, 200, and 730 µg), ascorbic acid (250, 500, 1,000, and 2,000 µg), cycloserine (500, 1,000, 2,000, and 3,000 µg), and bacitracin (1,000, 2,000, 3,000, and 4,000 µg). For the bottom images (**C and D**), compounds were spotted in the following ranges: polymyxin B (PxnB) (50, 100, and 200 µg), EDTA (10, 20, and 30 µg), ascorbic acid (50, 100, and 200 µg), and CTAB (100, 200, and 300 µg). Exposure times were optimized to capture the brightest luminescent signal on a whole agar plate.

The initial experiments were performed in liquid cultures (Fig. 1) but we also tested the ability of these compounds to induce biosensor expression on agar plates. In Fig. 3, bioluminescent rings of gene expression are observed on the edges of the inhibition zones after spotting the compounds in increasing amounts on agar. This expression pattern reveals a sensitive response to concentrations close to the minimal inhibitory concentration, which is the boundary between growth and non-growth. This pattern was similar in liquid cultures, where the highest biosensor responses were observed near but below the MIC (Table 2). EDTA, polymyxin B, and CTAB are strong inducers of both biosensors on agar, while cycloserine induced a moderate response, and bacitracin only shows induction of the *pmrF::lux* sensor at the highest amount tested (Fig. 2). The agar plate contains both strong and weak inducers, and the exposure time was optimized for the strong signal, which may be too short to visualize the signal from the weaker inducers. Almost all OM damaging compounds were detected by biosensors in liquid and agar cultures except ascorbic acid, which was not detected on agar, even though there is sufficient ascorbic acid to inhibit growth.

### Screening fungal supernatants for OM damage responsive biosensor activity

**Fig 4:**
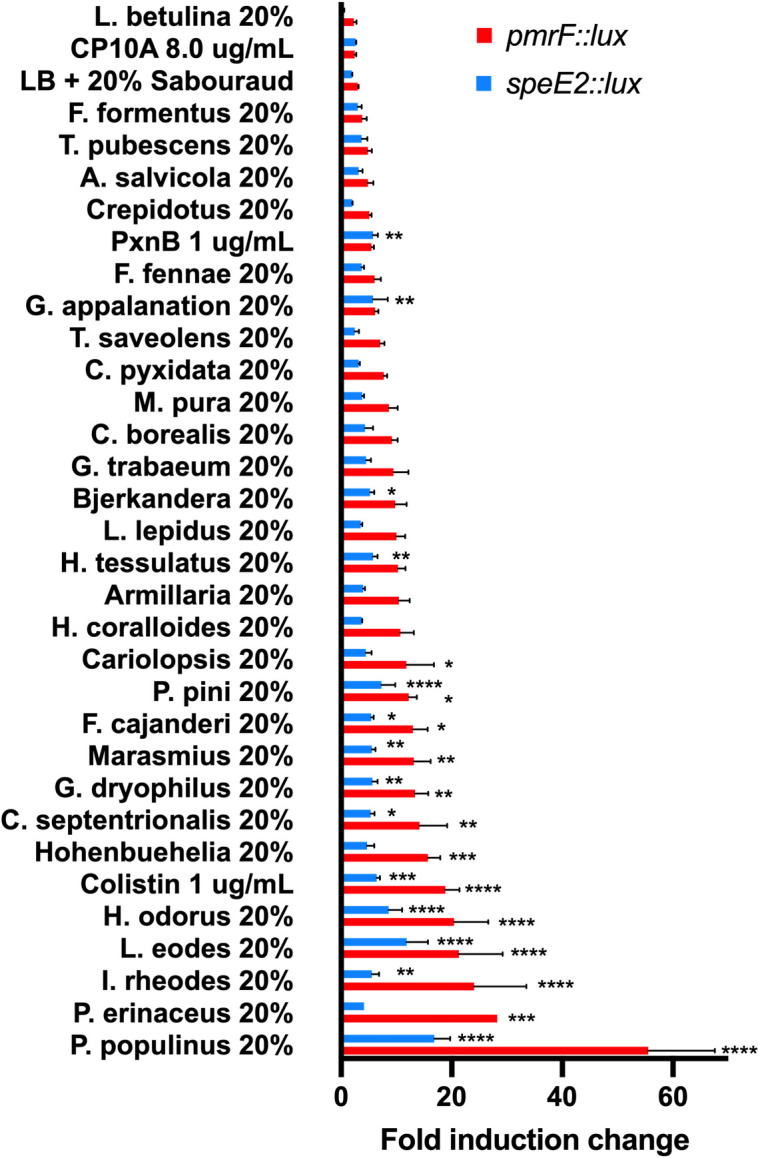
Screening of a collection of cell-free fungal supernatants for activity in the outer membrane damage-responsive biosensors *pmrF::lux* and *speE2::lux*. All supernatants were tested at 20% (vol/vol) in 96-well microplate assays for the ability to induce *lux* expression in the biosensor strains. The negative control was the addition of 20% sterile Sabouraud fungal growth medium to LB, and positive controls included the addition of antimicrobial peptides colistin, polymyxin B, or CP10A. Gene expression (CPS) was normalized to growth (OD_600_), and the fold induction change was calculated by dividing the gene expression with supernatant treatment by the expression with no treatment. The gene expression in liquid LB cultures was measured at the late log 7-hour time point. The values shown are the mean and standard error values of 3–5 replicates. All experiments were repeated at least three times. Significant differences compared with the untreated controls were determined by one-way ANOVA with the Dunnett’s multiple comparisons post-test (****P < 0.0001, ***P < 0.001, **P < 0.005, and *P < 0.05).

**Fig 5:**
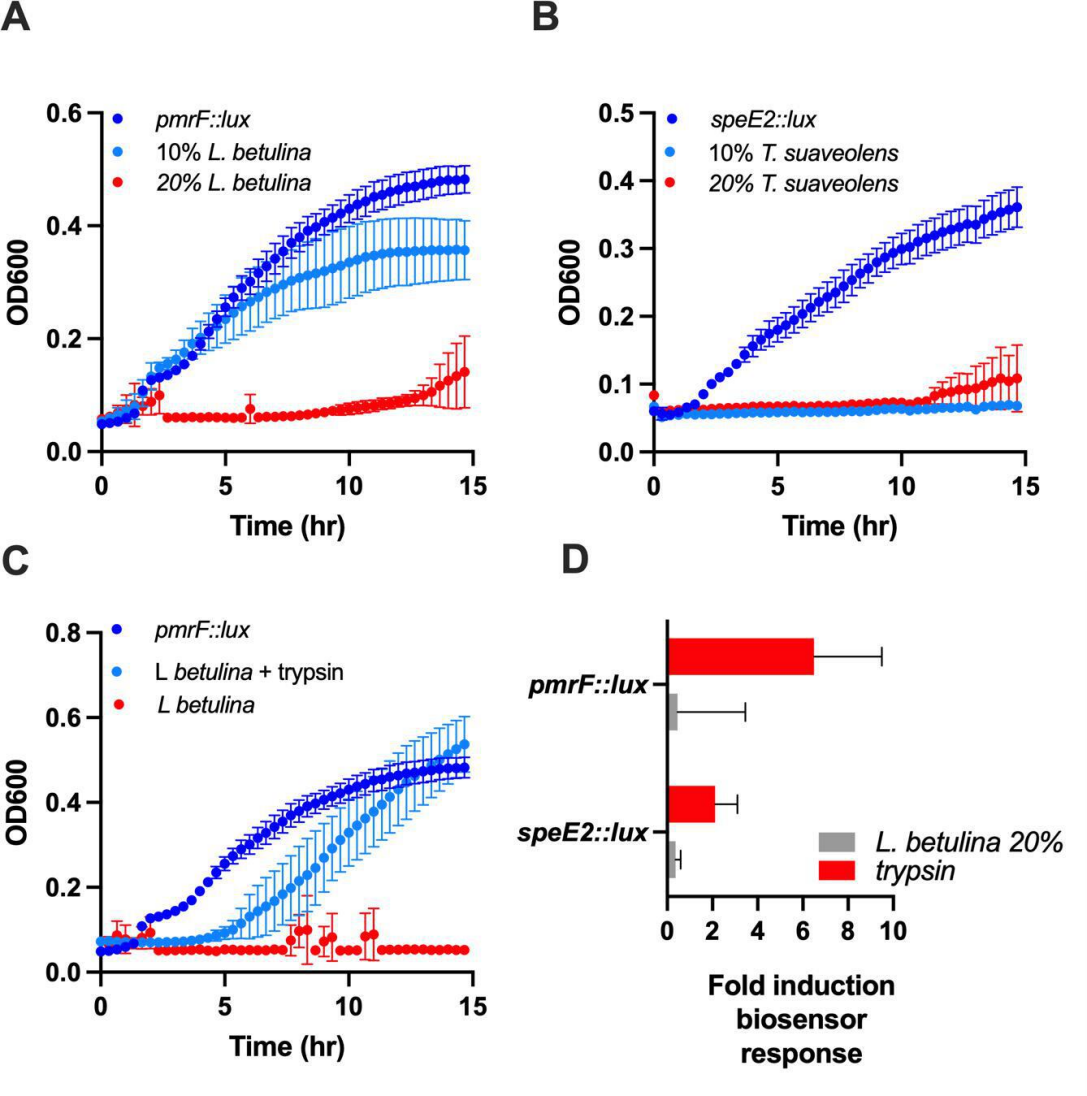
Cell-free culture supernatants from *L. betulina* and *T. suaveolens* inhibit *Pseudomonas aeruginosa* growth. Bacterial growth (OD_600_) of *pmrF::lux* or *speE2::lux* was measured in the presence of 10% and 20% vol/vol of fungal cell-free supernatants that were untreated (**A and B**). Trypsin digestion of *L. betulina* supernatant was performed before addition to bacterial cultures (light-blue) and compared with growth in control cultures (dark-blue) or supplementation with non-trypsin treated 20% supernatant (red) (**C**). (**D**) The effect on the *speE2::lux* and *pmrF::lux* biosensors of *L. betulina* supernatant with and without trypsin pretreatment. All values shown are the average of triplicates and the standard error. All experiments were performed at least three times.

Conventional methods of antimicrobial screening rely on the use of direct killing assays by natural products that are not sensitive enough to detect sub-lethal or dilute antimicrobial activities. Since fungi are known to produce numerous antibiotics, a collection of 29 fungal species (Table 1) was isolated in Alberta and screened using these biosensors to detect potential antimicrobial activity in the cell-free supernatants. Small volumes (20 µL) of ~80% of fungal supernatants caused a minimum fivefold induction or greater response of the *pmrF::lux* and *speE2::lux* biosensors, which was comparable to the known antimicrobial peptide polymyxin B (Fig. 4). Approximately 50% of the supernatants caused statistically significant induction responses from either biosensor, often with stronger responses than the positive control antimicrobial peptides polymyxin B and colistin (Fig. 4). Although the responses were generally higher in the *pmrF::lux* biosensor compared with *speE2::lux*, both sensors generally responded similarly to this panel of fungal cultures (Fig. 4). The supernatant of *Lenzites betulina* only caused a minimal induction of the *pmrF::lux* biosensor, but that is because this supernatant inhibited growth when present at 10%–20% (vol/vol) (Fig. 5).

### Fungal supernatants inhibit the growth of *Pseudomonas aeruginosa*

A few fungal supernatants inhibited the growth of the *P. aeruginosa* biosensors, suggesting the presence of conventional antimicrobial compounds. There was increasing growth inhibition with increasing concentrations of *L. betulina* supernatant (Fig. 5A), and only 10 µL (10% vol/vol) of *T. suaveolens* supernatant was needed to completely inhibit *Pseudomonas* growth (Fig. 5B). There was complete restoration of *Pseudomonas* growth after trypsin treatment of *L. betulina* supernatant (Fig. 5C), indicating the presence of an antimicrobial fungal protein. In addition, the biosensor responses increased after exposure to *L. betulina* supernatants that were trypsin treated and growth was restored (Fig. 5D).

### Biosensor-guided purification of antimicrobial activity in fungal supernatants

**Fig 6:**
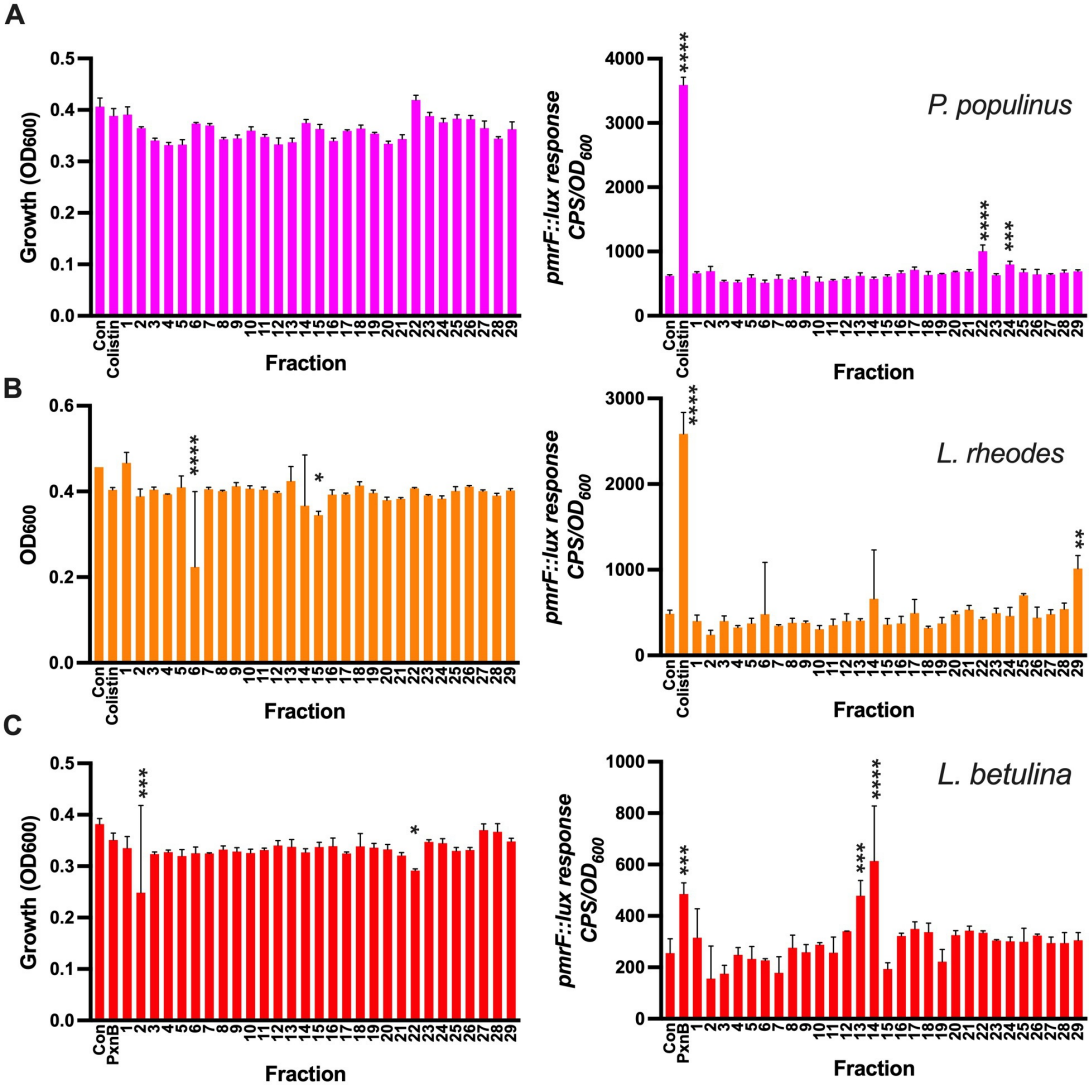
Biosensor-guided purification of active fractions containing biosensor activity from fungal supernatants. Fractions were collected and tested by adding (20% vol/vol) to the *pmrF::lux* biosensor grown in LB. The effects of the supernatants were tested on growth (OD_600_; left) and biosensor activity (CPS/OD_600_; right). Small volumes of fractionated supernatants (10–20 µL) were tested from (A) *P. populinus* (pink), (**B**) *L. rheodes* (orange), and (C) *L. betulina* supernatants (red). Negative controls had 20 µL of 50 mM ammonium bicarbonate fractionation buffer added to LB cultures, and the positive controls added sublethal amounts of polymyxin B or colistin. The values shown are the mean and standard error values of three replicates. Each fractionation experiment was performed once. Significant differences compared with the untreated controls (con) were determined by one-way ANOVA with the Dunnett’s multiple comparisons post-test (****P < 0.0001, ***P < 0.001, **P < 0.005, and *P < 0.05).

Fungal cultures were grown over a period of 8 weeks, and samples were collected and tested over time to determine the period of maximal activity by the biosensors (data not shown). Large volume cultures were then grown to those precise time points to maximize purification of the active component in these cultures using gel filtration chromatography. Fractions were collected and re-tested for the ability to inhibit growth or induce a *pmrF::lux* biosensor response. In fractions collected from *P. populinus* culture supernatants, which produced the strongest biosensor response (Fig. 4), biosensor activity was detected in fractions 22 and 24 (Fig. 6). In *L. rheodes* supernatants, fractions 6 and 15 caused growth inhibition and fraction 29 induced a biosensor response (Fig. 6). *L. betulina* supernatants were selected due to its antimicrobial activity (Fig. 5) and fractions 2 and 22 caused growth inhibition, while fractions 13 and 14 induced the biosensor responses, suggesting the presence of more than one antimicrobial compound (Fig. 6).

### Identification of an antimicrobial fungal, glycoside hydrolase protein

**Fig 7:**
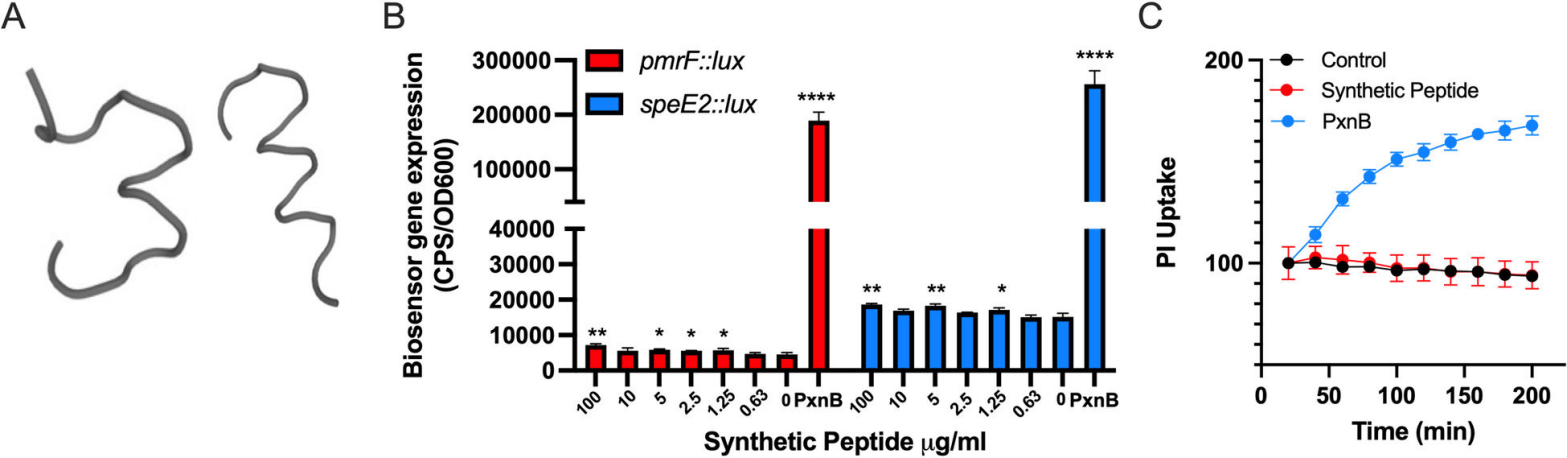
A synthetic peptide derived from a secreted *Lenzites betulina* glycoside hydrolase induces weak OM damage biosensor responses but does not disrupt the outer membrane. (**A**) The predicted alpha helical structure of the fungal peptide RADDTTVLSASGPGRN derived from a fungal glycoside hydrolase using PEP-FOLD^3^. (**B**) This synthetic peptide was tested for biosensor activity against both *pmrF::lux* and *speE2::lux*. The values shown are the mean of triplicates and the standard deviation at the maximum time point of gene expression (350 minutes). Significant differences were determined using unpaired *t*-test (*P < 0.05, **P < 0.01, and ****P < 0.0001). (**C**) Propidium iodide staining of *P. aeruginosa* PAO1 was used as a measure of OM damage and uptake of this membrane impermeable stain that binds DNA, where polymyxin B was used a positive control. The values shown are normalized to the control at time 0 and are the mean of triplicates and the standard deviation at each time point. All experiments were performed at least three times.

Using active fraction 14 from *L. betulina* supernatant fractionation (Fig. 6), a 7-kD protein band was excised from an SDS-PAGE gel and sent for LC-MS-MS protein identification. Peptides were identified with strong matches with a fungal protein that belongs to the glycoside hydrolase family, which includes hydrolytic enzymes such as lysozyme that degrade the bacterial cell wall. We ordered a synthetic peptide with the sequence RADDTTVLSASGPGRN derived from this fungal glycoside hydrolase because it also had a predicted alpha helical secondary structure (Fig. 7A), which is typical of antimicrobial peptides. Although this peptide did not have any antimicrobial activity (data not shown), it did have a very weak induction of the *pmrF::lux* biosensor (Fig. 7B). To determine if this peptide had a direct effect on disrupting the IM, we used a PI uptake assay as a measure of membrane damage. While the antimicrobial peptide polymyxin B caused PI uptake, the synthetic peptide derived from a fungal glycoside hydrolase did not disrupt the membrane and influence the uptake of the DNA binding dye PI (Fig. 7C). It was difficult to specifically identify the exact fungal protein or proteolytic fragment that was ultimately responsible for the antimicrobial activity produced by *L. betulina*.

### Screening herbal plant extracts for antimicrobial activity

**Fig 8:**
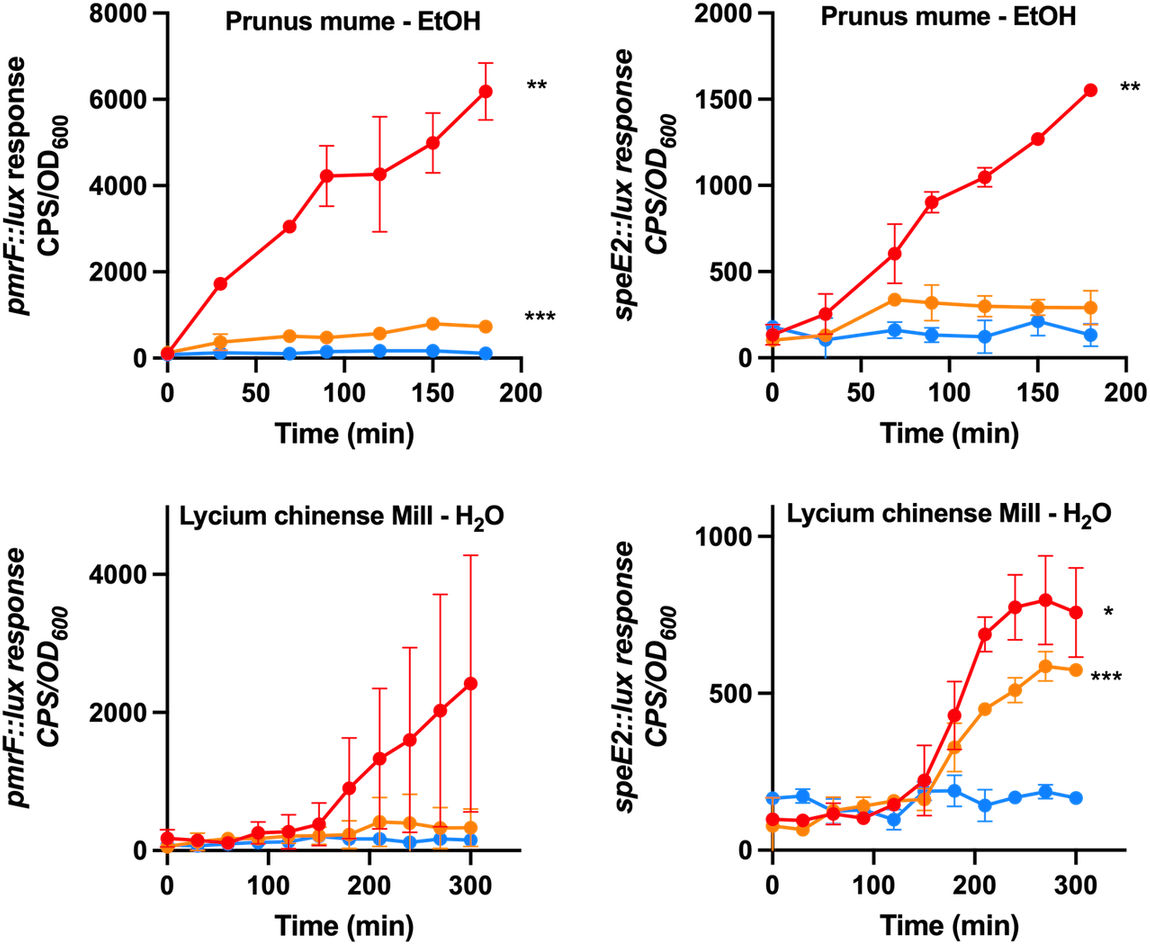
Chinese medicinal plant extracts induce the expression in the outer membrane damage-responsive biosensors. An ethanol extract of *Prunus mume* and a water extract of *Lycium Chinense* Mill plants were added at two concentrations to the *pmrF::lux* and *speE2::lux* biosensors grown in LB. The values shown are the average of duplicates and the standard deviation. Negative controls of LB cultures with no extracts are shown in blue, while orange and red represent extracts added at 1.5 mg/mL and 3 mg/mL, respectively. Significant differences were determined using unpaired *t*-test (*P < 0.05, **P < 0.01, and ***, *P* < 0.001).

A collection of 23 Chinese medicinal plants was assembled (Table 1), and both water and ethanol extracts of plant material were prepared. A total of 46 extracts were tested against the *pmrF::lux* and *speE2::lux* biosensors. Significant outer membrane disruption activity was detected in both sensors by 2/23 plants, including an ethanol (EtOH) extract of *Prunus mume* and a water (H_2_O) extract of *Lycium Chinense* Mill (Fig. 8). Biosensor activity increased with increasing amounts of extract material, with the highest biosensor responses occurred at total protein concentrations between 1.6 and 3 mg/mL.

### Screening a 400-member collection of drug-like compounds from the pathogen box

**Fig 9:**
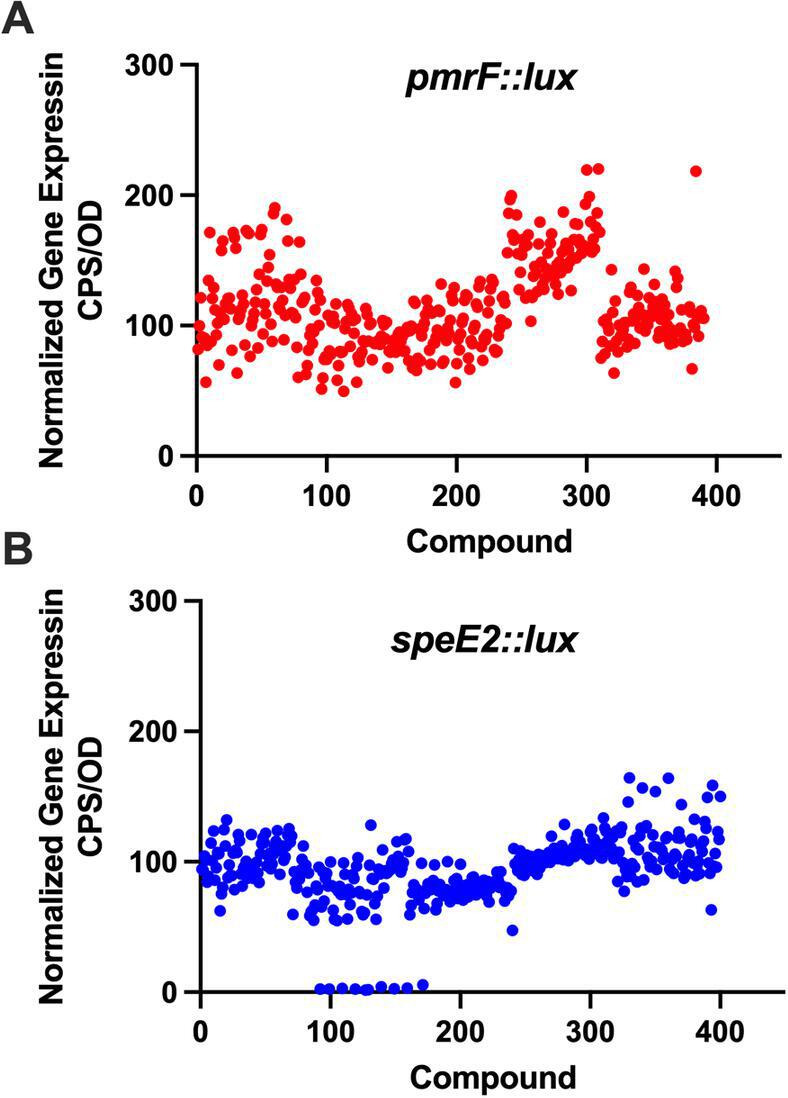
Screening of the pathogen box library for transcriptional induction of the *pmrF* and *speE2* promoters. All 400 compounds from the library were screened individually in 96-well microplates for induction of the (A) *pmrF::lux* and (B) *speE2::lux* biosensors, which are indicators of outer membrane disruption. All values shown reflect gene expression levels in the presence of 100 µM of each compound, and expression was normalized to 100% by comparing with the expression of negative LB media controls with no compound added. Gene expression values were taken at their maximum levels after 165 minutes of exposure.

This relatively small-sized drug library contains mainly compounds with activity against many tropical, parasitic diseases, as well as some bacterial and viral pathogens (18). To demonstrate the high-throughput and rapid screening of drug libraries with biosensors and to possibly identify drugs for repurposing, we screened all compounds against both the *pmr* and *spe* transcriptional *lux* reporters in search of compounds that induced the biosensor expression, suggestive of outer membrane damage. Almost all compounds had no effect on both biosensors, except for three which induced just above our minimum threshold of twofold induction of the *pmrF::lux* reporter (~200% of control gene expression levels) (Fig. 9). Given that the compounds were present at 100 µM, we considered this a weak response and did not pursue any further testing with these three compounds. There were also no compounds that inhibited *Pseudomonas* growth except for nine compounds from plate B that appeared to inhibit growth and/or gene expression (Fig. 9). However, eight of the nine compounds were within one column, and it was likely an experimental pipetting error that failed to inoculate that column of wells.

## DISCUSSION

Whole cell biosensors constructed using stress-inducible promoters have previously been used as tools to help identify the mechanism of antibiotic action, to detect antimicrobials in food or soil and in the context of antibiotic discovery (22 – 24). The first reported biosensor to detect bacterial outer membrane stress measures expression from the *fabA* gene, which encodes a 3-hydroxydecanoyl-ACP dehydrase that is required for fatty acid biosynthesis. The *fabA* promoter is induced under conditions of fatty acid starvation (cerulenin treatment) and by treatment with phenols and ethanol, which are known to disrupt membrane integrity (25). The Rcs (regulation of capsular polysaccharide) two-component system responds to disruptions to peptidoglycan assembly, defective lipoprotein trafficking, defects in the β-barrel assembly (BAM) system, and changes in LPS charge (26). In response to these various disruptions of the bacterial cell envelope, the *rprA* gene is upregulated, encoding a regulatory RNA that in turn controls stress-responsive regulatory proteins. The *rprA* promoter was cloned as a transcriptional reporter to the green fluorescent protein mNeonGreen, and this promoter was strongly induced by antimicrobial peptides, as well as slower, weaker responses to inhibitors of the BAM complex and lipoprotein maturation (26). This *rprA*-based reporter was proposed as a candidate high-throughput screening method for future discovery of envelope-disrupting antimicrobials (26).

Here, we propose a novel *pmr/spe* biosensor assay detection of compounds that cause outer membrane damage in the Gram-negative pathogen *Pseudomonas aeruginosa*, in contrast to numerous antimicrobials that target essential enzymes required for cell envelope synthesis (27). Other microplate-based permeability methods to assess outer membrane damage include dye assays, such as the NPN uptake method (28), or the leakage of cytoplasmic (29) or periplasmic-localized (30) β-galactosidase. The main advantages to the biosensor method include its high-throughput screening capacity, real-time measurement with no added substrate, sensitivity to sublethal antibiotic concentrations, a wide dynamic range and dose-dependent response to strong and weak inducers, the ability to specifically detect a diverse group of known OM-disrupting antimicrobials, as well as enzyme inhibitors that interfere with synthesis of the cell envelope, and assay conditions resembling normal growing conditions. In addition, the strains used here are both transcriptional reporters and insertional transposon mutants, which is important as the OM of these biosensor strains are more sensitive to OM disruption and killing by polymyxin B and aminoglycosides (2, 4, 10), which likely increases their sensitivity when compared with a wild-type background strain. Consistent with this application, these transcriptional *lux* fusions to *pmrF* and *speE2* were previously used to help characterize the bacterial membrane-targeting mechanism of novel, antimicrobial defensin peptides from plants (31). Given the response of these two membrane-protective systems to direct outer membrane threats (NETs, antimicrobial peptides, chelators, and detergents), we consider these genes as general membrane repair processes that support survival from membrane attack.

We applied this biosensor screen to identify antimicrobial and biosensor activity from fungal and plant natural products. In addition, the biosensor was useful in the purification of active fractions from fungal supernatants. We attempted to purify a fungal protein from *Lenzites betulina*, but the precise protein/peptide identification using mass spectrometry was limited due to the relative lack of fungal genomes in the databases. A peptide from this fungal protein was synthesized but had no antimicrobial activity and weakly induced the biosensors. We also attempted to isolate small molecules responsible for the biosensor activity in the *H. odorus* fungal supernatant, but the scale of purification was insufficient for complete structural identification (data not shown). The main limitation of this study was the inability to purify a single compound that acts on the outer membrane. Future work will be needed for large-scale purification, structure identification, and further characterization of potential new membrane-targeting compounds. It is also important to control for other conditions that induce the expression of these promoters, such as acid pH. The addition of fungal culture supernatants to LB medium for the biosensor assays did not change the neutral pH of the LB screening conditions.

The *pmr* and *spe* genes are induced under various, stressful environmental conditions; however, the specific two-component systems needed in these diverse conditions is not well understood. For example, under Mg^2+^ limiting and acidic pH 5.5 conditions, the PhoPQ and PmrAB systems are both required for the expression of the *pmr* operon (5, 6, 10), while the *spe* operon requires only the PmrAB two-component system under Mg^2+^ limiting and acidic pH 5.5 conditions (5, 10). These observations suggest a functional overlap or redundancy between the PhoPQ and PmrAB two-component systems. It is thought that the CprSR and ParSR two-component systems sense antimicrobial peptides directly, which leads to expression of the *pmr* and *spe* operons in response to antimicrobial peptide exposure, also suggestive of functional overlap. Future work will aim to determine which of these two-component environmental sensing systems are required to sense and respond to diverse outer membrane disrupting treatments.
